# Association between frailty and mortality among patients with accidental hypothermia: a nationwide observational study in Japan

**DOI:** 10.1186/s12877-021-02459-5

**Published:** 2021-09-25

**Authors:** Shuhei Takauji, Toru Hifumi, Yasuaki Saijo, Shoji Yokobori, Jun Kanda, Yutaka Kondo, Kei Hayashida, Junya Shimazaki, Takashi Moriya, Masaharu Yagi, Junko Yamaguchi, Yohei Okada, Yuichi Okano, Hitoshi Kaneko, Tatsuho Kobayashi, Motoki Fujita, Keiki Shimizu, Hiroyuki Yokota, Arino Yaguchi

**Affiliations:** 1 Department of Emergency Medicine, Asahikawa Medical University Hospital, 2-1, Midorigaoka higashi, Asahikawa, 078-8510 Japan; 2 Japan Association of Acute Medicine Heatstroke and Hypothermia Surveillance Committee, Tokyo, Japan; 3 Department of Emergency and Critical Care Medicine, St. Luke’s International Hospital, Tokyo, Japan; 4 Department of Social Medicine, Asahikawa Medical University, Asahikawa, Japan; 5 Department of Emergency and Critical Care Medicine, Nippon Medical School, Tokyo, Japan; 6 Department of Emergency Medicine, Teikyo University Hospital, Tokyo, Japan; 7 Department of Emergency and Critical Care Medicine, Juntendo University Urayasu Hospital, Chiba, Japan; 8 Department of Emergency Medicine, North Shore University Hospital, Northwell Health System, Manhasset, NY USA; 9 Department of Traumatology and Acute Critical Medicine, Osaka University Graduate School, Osaka, Japan; 10 Department of Emergency and Critical Care Medicine, Jichi Medical University Saitama Medical Center, Saitama, Japan; 11 Department of Emergency, Disaster and Critical Care Medicine, Showa University School of Medicine, Tokyo, Japan; 12 Department of Acute Medicine, Nihon University School of Medicine, Tokyo, Japan; 13 Department of Primary care and Emergency medicine, Graduate school of medicine, Kyoto University, Kyoto, Japan; 14 Department of Emergency Medicine, Japanese Red Cross Kumamoto Hospital, Kumamoto, Japan; 15 Emergency and Critical Care Center, Tokyo Metropolitan Tama Medical Center, Tokyo, Japan; 16 Department of Emergency and Critical Care Medicine, Aizu Chuo Hospital, Aizuwakamatsu, Japan; 17 Advanced Medical Emergency and Critical Care Center, Yamaguchi University Hospital, Ube, Japan; 18 Department of Critical Care and Emergency Medicine, Tokyo Women’s Medical University, Tokyo, Japan

**Keywords:** Accidental hypothermia, Frailty, Mortality, Activity of daily living, Rewarming rate

## Abstract

**Background:**

Frailty has been associated with a risk of adverse outcomes, and mortality in patients with various conditions. However, there have been few studies on whether or not frailty is associated with mortality in patients with accidental hypothermia (AH). In this study, we aim to determine this association in patients with AH using Japan’s nationwide registry data.

**Methods:**

The data from the Hypothermia STUDY 2018&19, which included patients of ≥18 years of age with a body temperature of ≤35 °C, were obtained from a multicenter registry for AH conducted at 120 institutions throughout Japan, collected from December 2018 to February 2019 and December 2019 to February 2020. The clinical frailty scale (CFS) score was used to determine the presence and degree of frailty. The primary outcome was the comparison of mortality between the frail and non-frail patient groups.

**Results:**

In total, 1363 patients were included in the study, of which 920 were eligible for the analysis. The 920 patients were divided into the frail patient group (*N* = 221) and non-frail patient group (*N* = 699). After 30-days of hospitalization, 32.6% of frail patients and 20.6% of non-frail patients had died (*p* < 0.001). Frail patients had a significantly higher risk of 90-day mortality (Hazard ratio [HR], 1.64; 95% confidence interval [CI], 1.25–2.17; *p* < 0.001). Based on the Cox proportional hazards analysis using multiple imputation, after adjustment for age, potassium level, lactate level, pH value, sex, CPK level, heart rate, platelet count, location of hypothermia incidence, and rate of tracheal intubation, the HR was 1.69 (95% CI, 1.25–2.29; *p* < 0.001).

**Conclusions:**

This study showed that frailty was associated with mortality in patients with AH. Preventive interventions for frailty may help to avoid death caused by AH.

**Supplementary Information:**

The online version contains supplementary material available at 10.1186/s12877-021-02459-5.

## Background

The incidence of accidental hypothermia (AH), which is defined by a body core temperature of < 35 °C [1], is low, however, severe hypothermia is associated with a high mortality rate [2, 3]. In severe hypothermia, intrinsic heat production by means of active movement and shivering, disappeared, leading to further progression in the decrease in body temperature. In Japan, which has a large elderly population, the mortality rate of all patients with AH is as high as 24.4–35% [4, 5], so effective prevention and intervention strategies are required.

Frailty is characterized by a decline in functioning across multiple physiological systems, accompanied by an increased vulnerability to stressors [6]. More recently, data have suggested that the presence of frailty places a person at increased risk of adverse outcomes, including hospitalization, and mortality [7]. Recently, frailty has also been noted in critically ill patients [8, 9]. However, to our knowledge, limited data exist regarding the relationship between AH and frailty. Clarification of the relationship between AH and frailty may provide useful insight for improving the prognosis of patients with hypothermia.

We hypothesized that frailty would be associated with a poor prognosis and mortality in patients with AH. For the purpose of verifying this hypothesis, we analyzed the Japan’s nationwide registry data on hypothermia.

## Material and methods

### Study design and setting

We performed a prospective, observational, multi-center registries of hypothermia: the Hypothermia STUDY 2018&2019. This study was conducted from December 2018 to February 2019 and December 2019 to February 2020, among a consortium of 120 academic and community medical centers from different geographic regions across Japan. The study has been approved by the Ethics Review Board of Teikyo University Hospital in Japan (Approval No: 17–090). The requirement for informed consent was waived due to the observational nature of the study by the Ethics Review Board of Teikyo University Hospital in Japan. In addition, the institutional review board of each hospital listed in the acknowledgements approved the study.

### Patient selection and data collection

The present study included consecutive patients whose body temperature, as measured by emergency medical services (EMS) or at the emergency department (ED), was < 35 °C. Patients of < 18 years of age were excluded. The following data were collected: age, sex, any pre-existing conditions, activities of daily living (ADL), lifestyle, location of hypothermia incidence, mechanism underlying hypothermia (acute medical illness [stroke, ischemic cardiac disease, infectious disease, malnutrition, arrhythmia, diabetes mellitus, renal disease, hypoglycemia, cardiac failure, endocrine disease and gastrointestinal disease], trauma [submersion, distress], alcohol intoxication, other [including drugs]), Charlson comorbidity index (CCI) [10], Glasgow coma scale (GCS) [11], Sequential Organ Failure Assessment (SOFA) score [12], laboratory data, temperature, blood pressure, heart rate, respiratory rate, cardiac arrest during pre-hospital, intubation, hospital length of stay, mortality, and Cerebral Performance Category (CPC) [13] score at 30 days after admission, and complications. The temperature was recorded as the core temperature from the rectum, urinary bladder, or esophagus if available; otherwise, the peripheral temperature from the axilla or ear was noted. The severity of hypothermia was classified according to the temperature as mild (35–32 °C), moderate (32–28 °C), or severe (< 28 °C) with reference to previous studies [1] [3].

The laboratory data included the pH value, potassium level, lactate level, platelet count, CPK level, BUN level, and creatinine level measured at the ED. The pH value in principle was evaluated by an arterial blood gas analysis, and the pH value measured using the venous blood gas was adjusted as described in a previous study [14]. In the present study, the patients who did not stay in a hospital, or in whom the length of hospital stay or body temperature was unknown or > 35 °C were excluded from the present analysis.

Complications during hospitalization were recorded and classified as arrhythmia, pneumonia, pancreatitis, electrolyte abnormality, or other. Pneumonia was defined as an obvious shadow on chest radiography or computed tomography (CT). Pancreatitis was defined as cases meeting at least two of the following conditions: 1) abdominal pain, 2) elevation of pancreatic enzyme levels in the blood, and 3) edema of the pancreas or peripancreatic effusion on ultrasound/CT.

The rewarming duration to target temperature was defined as the time interval between arrival at the ED and the moment at which the target temperature was reached. The rewarming rate (°C per hour) was defined as follows: (target temperature-temperature at ED) / the rewarming duration to target temperature.

Rewarming methods were divided into active external rewarming (warmed blanket, forced warm air, heating pad, and warmed bath) and active internal rewarming (warmed fluid infusion, lavage, hemodialysis, intravascular catheter, and extracorporeal membrane oxygenation [ECMO]).

### Definition of frailty

The clinical frailty scale (CFS) score was used to determine the presence and degree of frailty, as described previously [15]. The CFS score was determined using the activities of daily living and pre-existing conditions, as shown in our previous study [16]: CFS 1, very fit, defined as ADL 1 (independent) and CCI 0; CFS 2, well, defined as ADL 1 and CCI ≥1, or ADL 2 (sometimes out of the door) and CCI 0; CFS 3, well with treated comorbid disease, defined as ADL 2 and CCI 1–2; CFS 4, apparently vulnerable, defined as ADL 2 and CCI ≥3, or ADL 3 (indoors); CFS 5, mildly frail, defined as ADL 4 (almost needing assistance) and CCI ≤2; CFS 6, moderately frail, defined as ADL 4 and CCI ≥3; and CFS 7, severely frail, defined ADL 5 (needing total assistance). Patients were defined as frail if they had a CFS score of ≥5 before hospital admission.

### Outcome measures

Patient demographics and outcomes were compared between frail and non-frail patients. The primary outcome was the comparison of mortality between the frail and non-frail patient groups. The secondary outcomes were the comparisons of the length of intensive care unit (ICU) stay, hospital stay, CPC at 30 days after admission, and complications between the frail and non-frail patient groups. A favorable outcome was defined as a CPC of 1 or 2, whereas an unfavorable outcome was defined as a CPC 3–5.

### Data analyses

Data are expressed as the number (%), median (interquartile range) or the mean ± standard deviation, as appropriate. Intergroup comparisons were made using the Fisher’s exact test for categorical data and Mann-Whitney U test or Student’s *t*-test for continuous data. Ninety-day survival was calculated using a Kaplan-Meier curve and the difference in survival between frail and non-frail patients was determined using a log-rank test. Hazard ratios (HRs) and the corresponding 95% confidence intervals (CI) of the association between frailty and 90-day survival were derived using Cox proportional hazard survival models. The following covariates were included in the multivariable model based on the relevant literature [2, 3], or the consideration of clinically significant variables: age, sex, potassium level, lactate level, pH value, CPK level, heart rate, platelet count, location of hypothermia incidence, and rate of tracheal intubation. Missing data were managed with multiple imputation by chained equations [17, 18]. The variables included in the imputation model were those from the multivariable model. Twenty-five datasets were imputed with 10 iterations each. A Cox proportional hazards analysis was applied to the 25 imputed datasets, and final estimates were obtained by averaging the 25 estimates according to Rubin’s rules. Furthermore, a complete data set was used for the sensitivity analysis. We also performed a subgroup analysis with the exclusion of cases in which a warmed blanket or ECMO were applied. All tests were two-sided, and *P* values of < 0.05 were considered statistically significant. All statistical analyses were performed with EZR (Saitama Medical Center, Jichi Medical University, Saitama, Japan), a graphical user interface for the R software program. Multiple imputation was performed using the mice package in R (version, 4.0.3 R Foundation for Statistical Computing, Vienna, Austria).

## Results

**Fig. 1:**
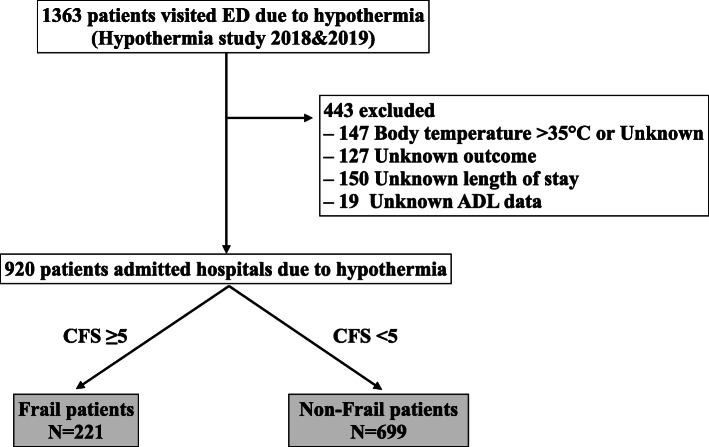
Flow chart of the enrolment of the study participants. Of the 1363 patients with hypothermia, 920 patients were enrolled, and 443 patients were excluded. The 920 patients were divided into the frail group (*N* = 221) and the non-frail group (*N* = 699). ED, emergency department; CFS, clinical frailty scale

Of the 1363 patients with hypothermia who were included in the Hypothermia STUDY 2018&2019, 443 were excluded from the present study because of unknown temperature or temperature > 35 °C (*N* = 147), unknown outcome (*N* = 127), unknown length of hospital stay (*N* = 150), or unknown ADL (*N* = 19). The remaining 920 patients were eligible for inclusion in the present analysis. A patient flow diagram is shown in Fig. 1. According to the CFS score, the 920 patients were divided into the frail patient group (*N* = 221) and the non-frail patient group (*N* = 699).

### Baseline characteristics of the study population

Supplemental Fig. 1 shows the age distribution of the patients included in the present study. The present population included only a few relatively young patients, with 81% of the total patients being ≥65 years old, and the median patient age being 79 years old. Table 1 shows the baseline characteristics of the study population and a comparison of the clinical characteristics of frail and non-frail patients. The non-frail patient group had a larger percentage of male in comparison to the frail patient group. The frail patient group was older, had higher CCI values, and included a higher percentage of AH cases that occurred indoors in comparison to the non-frail patient group. Regarding the mechanism of hypothermia, the rate of acute medical illness in the frail patient group was higher than that in the non-frail patient group.

**Table 1:** Baseline characteristics

	All patients	Missing	Frail	Non-Frail	*p*-value
	*n* = 920	n, (%)	*n* = 221	*n* = 699	
Age, years	79 (68–87)	0	85 (78–90)	77 (66–86)	< 0.001
Male	513 (55.8%)	0	105 (47.5%)	408 (58.4%)	< 0.001
Charlson comorbidity index^a^	1 (0–2) 1.2 ± 1.6	0	1 (0–2) 1.5 ± 1.7	1 (0–2) 1.1 ± 1.5	< 0.001
Severity					
SOFA total	6 (3–8)	71 (7.7)	6 (4–8)	5 (3–8)	0.286
Clinical Frailty Scale score	2 (1–4)	0	5 (5–7)	2 (1–3)	< 0.001
Lifestyle		10 (1.1)			< 0.001
Living alone	298 (32.4%)		39 (17.8%)	259 (37.5%)	
Not living alone	549 (59.7%)		144 (65.8%)	405 (58.6%)	
Homelessness	3 (0.3%)		0 (0.0%)	3 (0.4%)	
Nursing home	48 (5.2%)		34 (15.5%)	14 (2.0%)	
Unknown	12 (1.3%)		2 (0.9%)	10 (1.4%)	
Location of hypothermia incidence		24 (2.6)			< 0.001
Outdoor	218 (23.7%)		14 (6.5%)	204 (30%)	
Indoor	678 (73.7%)		203 (93.5%)	475 (70%)	
Hypothermia caused mechanism		57 (6.2)			< 0.001
Acute medical illness	465 (50.5%)		123 (60.6%)	342 (51.8%)	
Trauma, Submersion, and distress	126 (13.7%)		17 (8.4%)	109 (16.5%)	
Alcohol intoxication	41 (4.5%)		3 (1.5%)	38 (5.8%)	
Others (Unknown, drug)	231 (25.1%)		60 (29.6%)	171 (25.9%)	

*SOFA* Sequential Organ Failure Assessment; The data are expressed as the number (%), median (interquartile range) or mean ± standard deviation

^a^The values were presented as the median and 25th–75th percentile because the Charlson comorbidity index showed a skewed distribution. However, these values were the same in the frail and non-frail groups despite the Mann-Whitney U test showing significance, so the mean and standard deviation are shown as well

### Clinical and laboratory data

Among the 920 patients, the core body temperature was measured in 585 (63.6%). The clinical and laboratory data are presented in Table 2. There were no significant differences in the severity grade of temperature, blood pressure, respiratory rate, potassium level, creatinine level, or cardiac arrest during the pre-hospital period between frail and non-frail patients. Frail patients had a lower GCS, heart rate, lactate level, platelet count, CPK level, and rate of tracheal intubation in comparison to non-frail patients. The pH values of frail patients were significantly higher in comparison to non-frail patients.

**Table 2:** Clinical and laboratory data of the patients with hypothermia

	All patients	Missing	Frail	Non-Frail	*p*-value
	*n* = 920	n, (%)	*n* = 221	*n* = 699	
Temperature	30.6 (28.2–33.1)	0	30.6 (28.6–33.0)	30.6 (28.1–33.2)	0.810
Mild (35–32 °C)	348 (37.8%)		81 (36.7%)	267 (38.2%)	0.081
Moderate (32–28 °C)	360 (39.1%)		99 (44.8%)	261 (37.3%)	
Severe (< 28 °C)	212 (23.0%)		41 (18.6%)	171 (24.5%)	
GCS	10 (7–14)	50 (5.4)	10 (7–13)	11 (7–14)	< 0.001
Systolic BP (mmHg)	117 (90–146)	82 (8.9)	115 (90–143)	117 (91–147)	0.705
Diastolic BP (mmHg)	68 (51–86)	96 (10.4)	67 (51–82)	69 (51–88)	0.454
Heart rate	72 (53–90)	30 (3.3)	62 (48–82)	73 (56–92)	< 0.001
Respiratory rate	18 (15–22)	89 (9.7)	18 (15–21)	18 (15–23)	0.423
pH	7.30 (7.19–7.37)	61 (6.6)	7.32 (7.23–7.39)	7.29 (7.18–7.37)	< 0.001
Potassium (mEq/L)	4.2 (3.7–4.9)	7 (0.8)	4.3 (3.7–4.9)	4.2 (3.7–4.9)	0.674
Lactate (mmol/L)	3.5 (1.8–7.6)	121 (13.2)	2.3 (1.1–6.8)	3.8 (2.0–8.2)	< 0.001
Plt (× 10^4^/μL)	18.1 (12.6–24.3)	13 (1.4)	16.1 (10.9–22.3)	18.7 (13.3–24.7)	< 0.001
CPK (U/L)	347 (138–1239)	72 (7.8)	249 (104–617)	393 (150–1494)	< 0.001
BUN (mg/dL)	31.7 (19.3–55.0)	13 (1.4)	35.1 (22.0–57.0)	30.4 (18.5–54.2)	0.020
Creatinine (mg/dL)	1.1 (0.7–1.8)	14 (1.5)	1.1 (0.7–1.9)	1.1 (0.7–1.8)	0.736
CPA	62 (6.7%)	2 (0.2)	12 (5.5%)	50 (7.2%)	0.443
Intubation	157 (17.1%)	60 (6.5)	23 (11.4%)	134 (20.3%)	< 0.001

*GCS* Glasgow Coma Scale, *CPA* cardiopulmonary arrest

The data are expressed as the number (%), median (interquartile range)

### Primary outcome

**Fig. 2:**
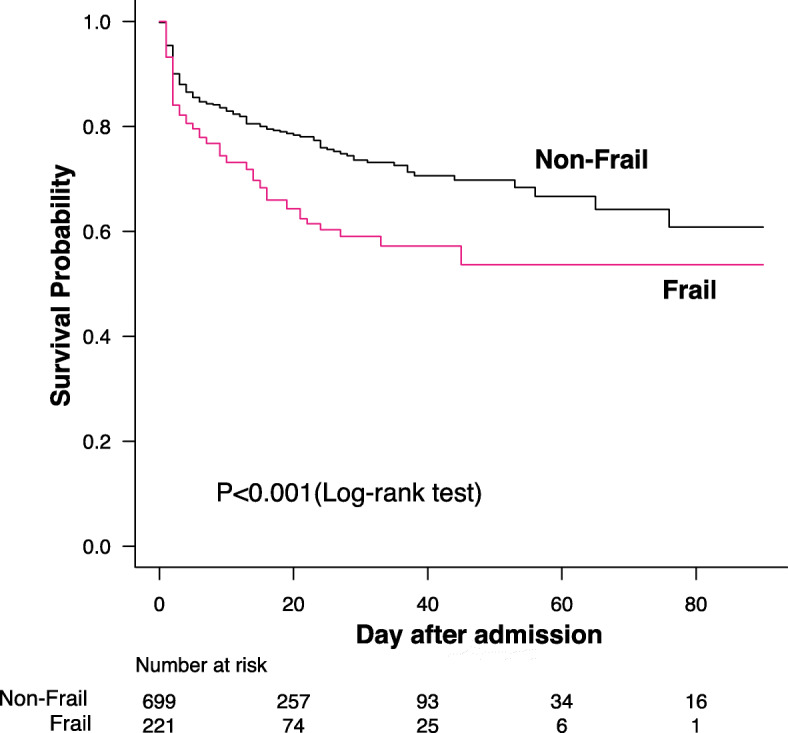
Probability of survival in patients with accidental hypothermia. Statistical comparison of survival of frail and non-frail patients, according to the Kaplan-Meier method

As shown in Table 3, the overall 30-day mortality rate was 23.5% (*N* = 216). After 30 days of hospitalization, 32.6% of frail patients and 20.6% of non-frail patients had died (*p* < 0.001). A survival time analysis revealed that there was significant difference between frail and non-frail patients (log-rank test *p* < 0.001) (Fig. 2). The results of the Cox proportional hazards analysis are summarized in Table 4. In the unadjusted analysis, frail patients had a significantly higher risk of 90-day mortality (Hazard ratio [HR], 1.64; 95% confidence interval [CI], 1.25–2.17; *p* < 0.001). Based on the Cox proportional hazards analysis using multiple imputation, after adjustment for age, potassium level, lactate level, pH value, sex, CPK level, heart rate, platelet count, location of hypothermia incidence, and rate of tracheal intubation, frail patients still had a significantly higher risk of 90-day mortality (Hazard ratio [HR], 1.69; 95% confidence interval [CI], 1.25–2.29; *p* < 0.001). A sensitivity analysis performed using the complete dataset of cases excluding cases with missing values (*N* = 679) confirmed the robustness of the results.

**Table 3:** Mortality, hospital length of stay, neurological score, and complications

	All patients	Frail	Non-Frail	*p*-value
	*n* = 920	*n* = 221	*n* = 699	
30-day mortality	216 (23.5%)	72 (32.6%)	144 (20.6%)	< 0.001
Length of stay at ICU	3 (2–6)	3 (2–5)	4 (2–7)	0.090
Length of stay at hospital	13 (4–27)	11 (3–23)	13 (4–29)	0.081
CPC at 30 days				< 0.001
good (1–2)	302	27 (20.0%)	275 (57.2%)	
poor (3–5)	314	108 (80.0%)	206 (42.8%)	
Complication				
Arrhythmia	22	6 (2.7%)	16 (2.3%)	0.800
Pneumonia	5	2 (0.9%)	3 (0.4%)	0.599
Pancreatitis	1	1 (0.5%)	0 (0%)	0.240
Electrolyte abnormalities	3	0 (0%)	3 (0.4%)	1.000
Coagulopathy	5	2 (0.9%)	3 (0.4%)	0.599
Other	10	6 (2.7%)	4 (0.6%)	0.016

*ICU* Intensive care unit, *CPC* Cerebral Performance Category

The data are expressed as the number (%), median (interquartile range)

**Table 4:** The comparison of mortality in frail and non-frail patients with hypothermia (multivariate Cox regression analysis)

Variable	HR	95% CI	*P*-value
Frail (Model 1)	1.64	1.25–2.17	< 0.001
Multiple imputation model (*N* = 920)			
Frail^a^ (Model 2)	1.69	1.25–2.29	< 0.001
Complete data set model (*N* = 679)			
Frail^b^ (Model 3)	1.45	1.01–2.09	0.043

*HR* Hazard ratio, *CI* Confidence interval

^a^After multiple imputation, adjusted for age, potassium, lactate, pH, sex, CPK, heart rate, platelet, location of hypothermia incidence, and rate of tracheal intubation

^b^Adjusted for age, potassium, lactate, pH, sex, CPK, heart rate, platelet, location of hypothermia incidence, and rate of tracheal intubation

### Secondary outcomes

Among the 920 total patients, the median length of ICU stay was 3 days, and the median length of hospital stay was 13 days. There was no significant difference in the length of stay at the ICU or hospital between frail and non-frail patients (Table 3). However, in the neurological assessment, frail patients showed a higher rate of patients with a worsened neurological score (CPC 3–5) at 30 days after admission in comparison to non-frail patients, while non-frail patients showed a significantly higher rate of patients with a favorable neurological outcome (CPC 1–2) in comparison to frail patients. There was no significant difference in the incidence of complications between the frail and non-frail patient groups.

### Rewarming method and rewarming rate

The rewarming method and rewarming rate are presented in Table 5. The rates of warmed blanket (*P* < 0.001) and ECMO (*P* = 0.039) use in the frail patient group were lower in comparison to the non-frail patient group. However, the other rewarming methods did not differ between the two groups to a statistically significant extent. The rewarming rate in frail patients was significantly slower than that in non-frail patients (*p* < 0.001).

**Table 5:** Rewarming method and rewarming rate

	Frail	Non-Frail	*p*-value
	*n* = 221	*n* = 699	
Rewarming method			
Active external rewarming			
Warmed blanket	73 (33.6%)	156 (22.8%)	< 0.001
Forced warm air	130 (59.9%)	381 (55.8%)	0.307
Heating pad	4 (1.8%)	34 (5.0%)	0.052
Warmed bath	4 (1.8%)	15 (2.2%)	1.000
Active internal rewarming			
Warmed fluid infusion	144 (66.4%)	459 (67.2%)	0.868
Lavage	4 (1.8%)	17 (2.5%)	0.797
Hemodialysis	2 (0.9%)	8 (1.2%)	1.000
Intravascular catheter	3 (1.4%)	14 (2.0%)	0.775
ECMO	3 (1.4%)	30 (4.4%)	0.039
Rewarming rate (°C/h)	0.96 (0.62–1.30)	1.13 (0.74–1.54)	< 0.001

*ECMO* Extracorporeal membrane oxygenation

The data are expressed as the number (%), median (interquartile range)

### Subgroup analyses

In the subgroup analysis with the exclusion of cases in which a warmed blanket or ECMO were applied, the rewarming rate in frail patients was still lower than that in non-frail patients (Supplemental Table 1).

## Discussion

The present nationwide study showed that frail patients with AH had a significantly higher risk of mortality in comparison to non-frail patients with AH, even after adjustment for important confounders. Additionally, the frail patient group included a higher rate of patients with a worsened neurological outcome in comparison to the non-frail patient group. The rewarming rate in frail patients was delayed in comparison to non-frail patients.

Recently, frailty has been shown to be associated with mortality and adverse outcomes in patients with various conditions [7], including patients with chronic obstructive pulmonary disease [19], patients with inflammatory bowel disease [20], patients with AIDS [21], patients awaiting liver transplantation [22], hip fracture patients [23] and patients undergoing elective vascular surgery [24], independent of chronological age. However, whether or not frailty is associated with mortality in patients with AH has not previously been investigated. The present nationwide study showed, for the first time, that frailty is an important prognostic factor in patients with AH.

Previous studies showed that prognostic factors in AH include the potassium level, pH value, lactate level, and age [2, 3, 25–27]. Although these factors may be useful for predicting the prognosis and selecting an appropriate rewarming intervention, these factors cannot be controlled and do not help improve the prognosis of patients with AH. However, in contrast to the other factors, frailty is a factor that can be avoided with preventive intervention [28] [29]. The reduction of frailty might consequently lead to a decrease in the number of deaths caused by AH.

The rewarming rate in frail patients was slower than that in non-frail patients. Although the rates at which ECMO or a warmed blanket were used in the frail patient group were lower in comparison to the non-frail patient group, the results were also similar in the subgroup analysis that excluded cases in which ECMO or a warmed blanket were used. The reasons for the difference in the rewarming rate may be as follows. It is hypothesized that intrinsic heat production by the patient, such as shivering thermogenesis, does not occur sufficiently in frail patients with AH, resulting in delayed rewarming. In the present study, the finding that the CPK level was lower in the frail patient group may support this mechanism. A previous study showed that a decreased rewarming rate in patients with AH is associated with a high risk of underlying infection [30] and mortality [31]. In recent years, many studies have shown that the prognosis of septic patients with hypothermia is poor [32–34]. For this reason, it has been pointed out that homeostatic dysfunction, such as immune dysfunction, is related to the poor prognosis of these patients [35, 36]. Although there was no significant difference in the occurrence of infectious complications between the frail and non-frail patient groups in the present study, a similar mechanism may be responsible for the relationship between frailty and a poor prognosis in patients with AH. On the other hand, the results of this study could not clarify whether or not the rapid rewarming using invasive internal rewarming methods will reduce mortality and improve the prognosis of frail patients with AH. Thus, further studies are needed to address this problem.

In our previous study, we found that frail patients with AH showed prolonged hospitalization [16]. However, in this study, there was no significant difference in the length of hospital stay between the frail and non-frail patient groups. The reasons are as follows: the previous study excluded patients who died within 30 days, whereas the present study included these patients. The rate of early mortality within 30 days was higher in the frail group than in the non-frail group. As a result, the length of hospital stay in the frail group was shorter than that in the non-frail group, although the difference was not statistically significant.

A previous study showed that, among ICU patients requiring mechanical ventilation, the presence of frailty increased the likelihood of short-term mortality, and that these findings might play a role in informed shared decision-making with patients and families prior to the provision of mechanical ventilation [37]. In this study, the rate of tracheal intubation was lower among frail patients than among non-frail patients. This may be because these patients and their families did not wish to receive invasive treatment with intubation and ventilation.

Regarding complications, previous studies have reported that the incidence of complications is higher in frail patients [7]. However, in this study, the incidence of complications in the frail and non-frail patient groups did not differ to a statistically significant extent. The complications defined in this study (arrhythmia, pneumonia, pancreatitis, electrolyte abnormality and coagulopathy) occurred infrequently, which may have contributed to the lack of a significant difference.

The present study was associated with some limitations. First, we used the CFS score, which was calculated based on ADL and the CCI to determine frailty, while the standard tools for the diagnosis of frailty are the frailty index [38] or frailty phenotype [39]. Therefore, it remains to be verified whether the diagnosis of frailty in this study was accurate. In this regard, a comparative study regarding the accuracy of the CFS score is currently in progress [40]. Second, there were numerous missing data in relation to the rewarming rate. However, the volume of data including in this nationwide study was sufficient; thus, the results are considered robust. Third, we could not to determine the rewarming rate according to individual rewarming methods, because several rewarming methods were used in combination. Finally, this study was based on the findings of registry data on hypothermia, and it did not include any data that was related to frail research, such as ADL after a long-term follow-up. Therefore, further studies will be needed to investigate the long-term ADL of frail patients with AH.

## Conclusions

This study found that, after adjustment for multiple factors, mortality in frail patients with AH was higher than that in non-frail patients with AH. According to the neurological outcome after 30 days, the percentage of patients with a poor prognosis in the frail patient group was higher than that in the non-frail patient group. It is important to recognize that frail patients with AH are at risk for more severe hypothermia.

## Supplementary Information

**Additional file 1 **: **Supplemental Fig. 1**. The age distribution of patients with accidental hypothermia. Of the 920 patients, 746 (81%) were ≥ 65 years old.

**Additional file 2 **: **Supplemental Table 1**. The rewarming rate in the sub-analysis after excluding cases in which ECMO or a warmed blanket was used.

## Data Availability

The datasets and analyzed during the current study are available from the corresponding author on reasonable request.

## Abbreviations

AH: Accidental hypothermia
CFS: Clinical frailty scale
HR: Hazard ratio
CI: Confidence interval
EMS: Emergency medical services
ED: Emergency department
ADL: Activities of daily living
CCI: Charlson comorbidity index
GCS: Glasgow coma scale
SOFA: Sequential Organ Failure Assessment
CPC: Cerebral Performance Category
ECMO: Extracorporeal membrane oxygenation
ICU: Intensive care unit
CT: Computed tomography

## References

[1] Brown DJ, Brugger H, Boyd J, Paal P (2012). Accidental hypothermia. N Engl J Med.

[2] Silfvast T, Pettilä V (2003). Outcome from severe accidental hypothermia in southern Finland—a 10-year review. Resuscitation.

[3] van der Ploeg GJ, Goslings JC, Walpoth BH, Bierens JJ (2010). Accidental hypothermia: rewarming treatments, complications and outcomes from one university medical Centre. Resuscitation.

[4] Yokota H (2013). The clinical characteristics of hypothermic patients in the winter of Japan-the final report of hypothermia STUDY 2011. J Japan Assoc Acute Med.

[5] Matsuyama T, Morita S, Ehara N, Miyamae N, Okada Y, Jo T, et al. Characteristics and outcomes of accidental hypothermia in Japan: the J-point registry. Emerg Med J. 2018. 10.1136/emermed-2017-207238. 10.1136/emermed-2017-207238 29886414

[6] Clegg A, Young J, Iliffe S, Rikkert MO, Rockwood K (2013). Frailty in elderly people. Lancet (London, England).

[7] Hoogendijk EO, Afilalo J, Ensrud KE, Kowal P, Onder G, Fried LP (2019). Frailty: implications for clinical practice and public health. Lancet (London, England).

[8] Silva-Obregon JA, Quintana-Diaz M, Saboya-Sanchez S, Marian-Crespo C, Romera-Ortega MA, Chamorro-Jambrina C, Estrella-Alonso A, Andres-Esteban EM (2020). Frailty as a predictor of short- and long-term mortality in critically ill older medical patients. J Crit Care.

[9] Muscedere J, Waters B, Varambally A, Bagshaw SM, Boyd JG, Maslove D, Sibley S, Rockwood K (2017). The impact of frailty on intensive care unit outcomes: a systematic review and meta-analysis. Intensive Care Med.

[10] Charlson ME, Pompei P, Ales KL, MacKenzie CR (1987). A new method of classifying prognostic comorbidity in longitudinal studies: development and validation. J Chronic Dis.

[11] Teasdale G, Maas A, Lecky F, Manley G, Stocchetti N, Murray G (2014). The Glasgow coma scale at 40 years: standing the test of time. Lancet Neurol.

[12] Vincent JL, de Mendonça A, Cantraine F, Moreno R, Takala J, Suter PM, Sprung CL, Colardyn F, Blecher S (1998). Use of the SOFA score to assess the incidence of organ dysfunction/failure in intensive care units: results of a multicenter, prospective study. Working group on “sepsis-related problems” of the European Society of Intensive Care Medicine. Crit Care Med.

[13] A randomized clinical trial of calcium entry blocker administration to comatose survivors of cardiac arrest: Design, methods, and patient characteristics. Control Clin Trials. 1991;12(4):525–45. 10.1016/0197-2456(91)90011-a 1657528

[14] Bloom BM, Grundlingh J, Bestwick JP, Harris T (2014). The role of venous blood gas in the emergency department: a systematic review and meta-analysis. Eur J Emerg Med.

[15] Rockwood K, Song X, MacKnight C, Bergman H, Hogan DB, McDowell I, Mitnitski A (2005). A global clinical measure of fitness and frailty in elderly people. CMAJ.

[16] Takauji S, Hifumi T, Saijo Y, Yokobori S, Kanda J, Kondo Y, Hayashida K, Shimizu K, Yokota H, Yaguchi A (2021). Accidental hypothermia: factors related to a prolonged hospital stay – a nationwide observational study in Japan. Am J Emerg Med.

[17] Chevret S, Seaman S, Resche-Rigon M (2015). Multiple imputation: a mature approach to dealing with missing data. Intensive Care Med.

[18] White IR, Royston P, Wood AM (2011). Multiple imputation using chained equations: issues and guidance for practice. Stat Med.

[19] Marengoni A, Vetrano DL, Manes-Gravina E, Bernabei R, Onder G, Palmer K (2018). The relationship between COPD and frailty: a systematic review and Meta-analysis of observational studies. Chest.

[20] Faye AS, Wen T, Soroush A, Ananthakrishnan AN, Ungaro R, Lawlor G, et al. Increasing prevalence of frailty and its association with readmission and mortality among hospitalized patients with IBD. Dig Dis Sci. 2021. 10.1007/s10620-020-06746-w. 10.1007/s10620-020-06746-w PMC8493658 33385264

[21] Guaraldi G, Malagoli A, Theou O, Brothers TD, Wallace L, Torelli R, Mussini C, Sartini S, Kirkland SA, Rockwood K (2017). Correlates of frailty phenotype and frailty index and their associations with clinical outcomes. HIV Med.

[22] Sinclair M, Poltavskiy E, Dodge JL, Lai JC (2017). Frailty is independently associated with increased hospitalisation days in patients on the liver transplant waitlist. World J Gastroenterol.

[23] Schuijt HJ, Morin ML, Allen E, Weaver MJ. Does the frailty index predict discharge disposition and length of stay at the hospital and rehabilitation facilities? Injury. 2021;52(6):1384–89. 10.1016/j.injury.2021.01.004. 10.1016/j.injury.2021.01.004 33478798

[24] Banning LBD, El Moumni M, Visser L, van Leeuwen BL, Zeebregts CJ, Pol RA. Frailty leads to poor long-term survival in patients undergoing elective vascular surgery. J Vasc Surg. 2021;73(6):2132–9.e2. 10.1016/j.jvs.2020.10.088. 10.1016/j.jvs.2020.10.088 33387657

[25] Elbaz G, Etzion O, Delgado J, Porath A, Talmor D, Novack V (2008). Hypothermia in a desert climate: severity score and mortality prediction. Am J Emerg Med.

[26] Schaller MD, Fischer AP, Perret CH (1990). Hyperkalemia. A prognostic factor during acute severe hypothermia. Jama.

[27] Okada Y, Matsuyama T, Morita S, Ehara N, Miyamae N, Jo T, Sumida Y, Okada N, Kitamura T, Iiduka R (2019). Prognostic factors for patients with accidental hypothermia: a multi-institutional retrospective cohort study. Am J Emerg Med.

[28] de Labra C, Guimaraes-Pinheiro C, Maseda A, Lorenzo T, Millan-Calenti JC (2015). Effects of physical exercise interventions in frail older adults: a systematic review of randomized controlled trials. BMC Geriatr.

[29] Losa-Reyna J, Baltasar-Fernandez I, Alcazar J, Navarro-Cruz R, Garcia-Garcia FJ, Alegre LM, Alfaro-Acha A (2019). Effect of a short multicomponent exercise intervention focused on muscle power in frail and pre frail elderly: a pilot trial. Exp Gerontol.

[30] Delaney KA, Vassallo SU, Larkin GL, Goldfrank LR (2006). Rewarming rates in urban patients with hypothermia: prediction of underlying infection. Acad Emerg Med.

[31] Watanabe M, Matsuyama T, Morita S, Ehara N, Miyamae N, Okada Y, Jo T, Sumida Y, Okada N, Nozawa M, Tsuruoka A, Fujimoto Y, Okumura Y, Kitamura T, Ohta B (2019). Impact of rewarming rate on the mortality of patients with accidental hypothermia: analysis of data from the J-point registry. Scand J Trauma Resusc Emerg Med.

[32] Marik PE, Zaloga GP (2000). Hypothermia and cytokines in septic shock. Norasept II study investigators. North American study of the safety and efficacy of murine monoclonal antibody to tumor necrosis factor for the treatment of septic shock. Intensive Care Med.

[33] Kushimoto S, Gando S, Saitoh D, Mayumi T, Ogura H, Fujishima S, Araki T, Ikeda H, Kotani J, Miki Y (2013). The impact of body temperature abnormalities on the disease severity and outcome in patients with severe sepsis: an analysis from a multicenter, prospective survey of severe sepsis. Critical care (London, England).

[34] Kushimoto S, Abe T, Ogura H, Shiraishi A, Saitoh D, Fujishima S, Mayumi T, Hifumi T, Shiino Y, Nakada TA, Tarui T, Otomo Y, Okamoto K, Umemura Y, Kotani J, Sakamoto Y, Sasaki J, Shiraishi SI, Takuma K, Tsuruta R, Hagiwara A, Yamakawa K, Masuno T, Takeyama N, Yamashita N, Ikeda H, Ueyama M, Fujimi S, Gando S, JAAM Focused Outcome Research on Emergency Care for Acute respiratory distress syndrome, Sepsis and Trauma (FORECAST) Group (2019). Impact of body temperature abnormalities on the implementation of Sepsis bundles and outcomes in patients with severe Sepsis: a retrospective sub-analysis of the focused outcome research on emergency Care for Acute Respiratory Distress Syndrome, Sepsis and trauma study. Crit Care Med.

[35] Drewry AM, Fuller BM, Skrupky LP, Hotchkiss RS (2015). The presence of hypothermia within 24 hours of sepsis diagnosis predicts persistent lymphopenia. Crit Care Med.

[36] Drewry AM, Ablordeppey EA, Murray ET, Dalton CM, Fuller BM, Kollef MH, Hotchkiss RS (2018). Monocyte function and clinical outcomes in febrile and afebrile patients with severe Sepsis. Shock.

[37] Fernando SM, McIsaac DI, Rochwerg B, Bagshaw SM, Muscedere J, Munshi L, Ferguson ND, Seely AJE, Cook DJ, Dave C (2019). Frailty and invasive mechanical ventilation: association with outcomes, extubation failure, and tracheostomy. Intensive Care Med.

[38] Rockwood K, Mitnitski A (2007). Frailty in relation to the accumulation of deficits. J Gerontol A Biol Sci Med Sci.

[39] Fried LP, Tangen CM, Walston J, Newman AB, Hirsch C, Gottdiener J, Seeman T, Tracy R, Kop WJ, Burke G, McBurnie MA (2001). Frailty in older adults: evidence for a phenotype. J Gerontol A Biol Sci Med Sci.

[40] Leahy A, O'Connor M, Condon J, Heywood S, Shanahan E, Peters C, Galvin R (2021). Diagnostic and predictive accuracy of the clinical frailty scale among hospitalised older medical patients: a systematic review and meta-analysis protocol. BMJ Open.

