# Supplementary material for: Association between frailty and mortality among patients with accidental hypothermia: a nationwide observational study in Japan
Source: BMC Geriatr. 2021 Sep 25;21:507. doi: 10.1186/s12877-021-02459-5 (PMC8466946; doi:10.1186/s12877-021-02459-5)
Supplement: Supplementary file 1 — Additional file 1 : Supplemental Fig. 1. The age distribution of patients with accidental hypothermia. Of the 920 patients, 746 (81%) were ≥ 65 years old. [file 12877_2021_2459_MOESM1_ESM.pdf]

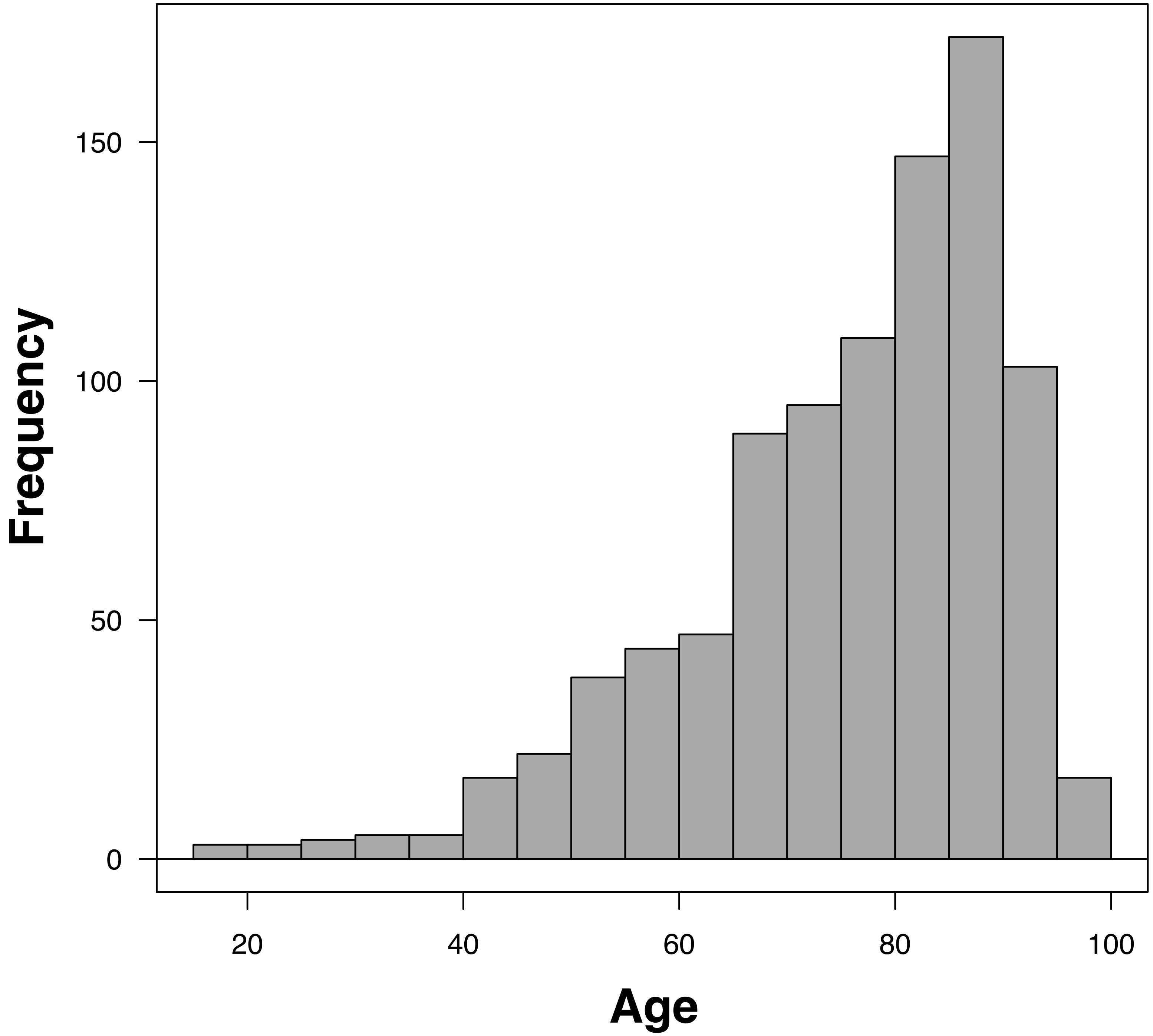

Supplemental Figure 1. The age distribution of patients with accidental hypothermia

Of the 920 patients, 746 (81%) were ≥ 65 years old.
