# Supplementary material for: Association between frailty and mortality among patients with accidental hypothermia: a nationwide observational study in Japan
Source: BMC Geriatr. 2021 Sep 25;21:507. doi: 10.1186/s12877-021-02459-5 (PMC8466946; doi:10.1186/s12877-021-02459-5)
Supplement: Supplementary file 2 — Additional file 2 : Supplemental Table 1. The rewarming rate in the sub-analysis after excluding cases in which ECMO or a warmed blanket was used. [file 12877_2021_2459_MOESM2_ESM.docx]

| Supplemental Table 1. The rewarming rate in the sub-analysis after excluding cases in which ECMO or a warmed blanket was used | | | |
| --- | --- | --- | --- |
|  |  |  |  |
|  | Frail | Non-Frail | p-value |
|  | n=145 | n=515 |  |
| Rewarming rate (°C/h) | 0.97 (0.68 – 1.35) | 1.21 (0.78 – 1.55) | 0.011 |
| ECMO, extracorporeal membrane oxygenation The data are expressed as a median (interquartile range). | | |  |
